# Pharmaceutical Therapies for Necroptosis in Myocardial Ischemia–Reperfusion Injury

**DOI:** 10.3390/jcdd10070303

**Published:** 2023-07-17

**Authors:** Yinchang Zhang, Yantao Zhang, Jinlong Zang, Yongnan Li, Xiangyang Wu

**Affiliations:** Department of Cardiac Surgery, Lanzhou University Second Hospital, Lanzhou University, Lanzhou 730030, China; zychdx@163.com (Y.Z.); 15922023525@163.com (Y.Z.); zjl_jordon@163.com (J.Z.)

**Keywords:** necroptosis, myocardial ischemia–reperfusion, RIPK1, RIPK3, MLKL, pharmaceutical therapies

## Abstract

Cardiovascular disease morbidity/mortality are increasing due to an aging population and the rising prevalence of diabetes and obesity. Therefore, innovative cardioprotective measures are required to reduce cardiovascular disease morbidity/mortality. The role of necroptosis in myocardial ischemia–reperfusion injury (MI–RI) is beyond doubt, but the molecular mechanisms of necroptosis remain incompletely elucidated. Growing evidence suggests that MI–RI frequently results from the superposition of multiple pathways, with autophagy, ferroptosis, and CypD-mediated mitochondrial damage, and necroptosis all contributing to MI–RI. Receptor-interacting protein kinases (RIPK1 and RIPK3) as well as mixed lineage kinase domain-like pseudokinase (MLKL) activation is accompanied by the activation of other signaling pathways, such as Ca^2+^/calmodulin-dependent protein kinase II (CaMKII), NF-κB, and JNK-Bnip3. These pathways participate in the pathological process of MI–RI. Recent studies have shown that inhibitors of necroptosis can reduce myocardial inflammation, infarct size, and restore cardiac function. In this review, we will summarize the molecular mechanisms of necroptosis, the links between necroptosis and other pathways, and current breakthroughs in pharmaceutical therapies for necroptosis.

## 1. Introduction

With the aging of the population, myocardial infarction has become the leading cause of mortality globally, posing a serious threat to human physical and mental health. Acute myocardial infarction (AMI) is a serious and common medical emergency that causes irreversible damage to the heart [1]. Most patients with AMI have a history of coronary artery disease, which leads to atherosclerotic plaque rupture, platelet aggregation, thrombus formation, and eventual blockage of the coronary lumen. The fundamental principles of therapy are the timely restoration of coronary blood flow and the decrease of myocardial infarct size. Reperfusion therapy, whether direct coronary intervention or thrombolytic therapy, has become a relatively standard and mature treatment. However, in the past few years, it has been observed that myocardial reperfusion may cause more myocardial cell death, and that reperfusion itself restores the supply of oxygen and nutrients to myocardial cells, saving the ischemic myocardium while causing additional myocardial injury, known as MI–RI. Studies in animal models have shown that reperfusion injury accounts for 25–50% of eventual myocardial infarction [2]. The series of pathophysiological events following reperfusion include the excessive release of intracellular Ca^2+^, impairment of the mitochondrial electron transport chain, massive production of reactive oxygen species (ROS), neutrophil recruitment, and inflammation, which can lead to irreversible loss of cardiomyocytes. This results in several fatal consequences, such as arrhythmias, fibrosis, and ventricular systolic and diastolic dysfunction [3]. Preventing or mitigating MI–RI has emerged as one of the most recent hot topics in cardiovascular disease research.

Many studies have investigated the pathogenesis of MI–RI, which is associated with oxidative stress, intracellular calcium overload, impaired energy metabolism [4,5], endoplasmic reticulum stress, apoptosis, necroptosis [6], autophagy [7,8], pyroptosis [9,10], and ferroptosis [11,12,13]. In addition, these mechanisms are interrelated and may contribute directly or indirectly to the exacerbation of cell death. Most previous studies on cardiomyocyte death have focused on apoptosis, whereas necrosis is considered a passive, unregulated process that occurs in response to severe pathological stress and is characterized by cell swelling, rupture of the cell membranes and organelles, and an efflux of cell contents [14]. Recent studies have questioned the role of apoptosis as a major contributor to cardiomyocyte loss during I/R injury [15]. Necroptosis, a form of cell death similar to the necrotic phenotype, has been identified in cardiac pathology. It has been reported that the rate of myocardial cell death by necroptosis is higher than that of apoptosis during the course of heart failure [16], and that I/R-induced myocardial cell death may account for up to 50% of the final myocardial infarct area [17]. Necroptosis is thought to play a role in cardiovascular disease by driving inflammation and inflammasome activation, as well as causing cell death [18]. It was found that ischemic preconditioning and post-treatment attenuates MI–RI-induced necroptosis [19], with inhibitors that have been proven to have cardioprotective effects. Here, we review the current information on the regulatory mechanisms of necroptosis and the involvement of its pharmacological agents in MI–RI.

## 2. Roles of Necroptosis during Myocardial Ischemia or I/R Injury

Necroptosis is a newly discovered form of regulated cellular necrosis with dual characteristics of necrosis and apoptosis. In terms of cell morphology, necroptosis is mainly characterized by cell swelling and cytoplasmic membrane rupture [20,21]. Both necroptosis and apoptosis are forms of cell death that may be regulated. Necroptosis is a type of caspase-independent programmed cell necrosis that is also negatively regulated by caspase-8 and is triggered by death receptors (such as tumor necrosis factor receptor 1, TNFR1). It also requires the kinase activities of RIPK1 and RIPK3. Necroptosis is often accompanied by the release of damage-associated molecular patterns (DAMP) and cytokines, which induce an immune inflammatory response and exacerbate tissue damage [22,23]. Necroptosis plays a key role in the extent of myocardial cell loss after MI–RI and the development of poor postischemic remodeling and cardiac dysfunction. Recently, there has been growing evidence that necroptosis leads to irreversible loss of cardiomyocytes, ventricular remodeling, and post-MI cardiac dysfunction [24,25]. Necroptosis can be induced by a variety of stimuli, including death ligands l, Toll-like receptors, and certain pathogens. Tumor necrosis factor (TNF)-induced necroptosis is the most common pathogen, mediated mainly by RIPK1 and RIPK3. 

**Figure 1:**
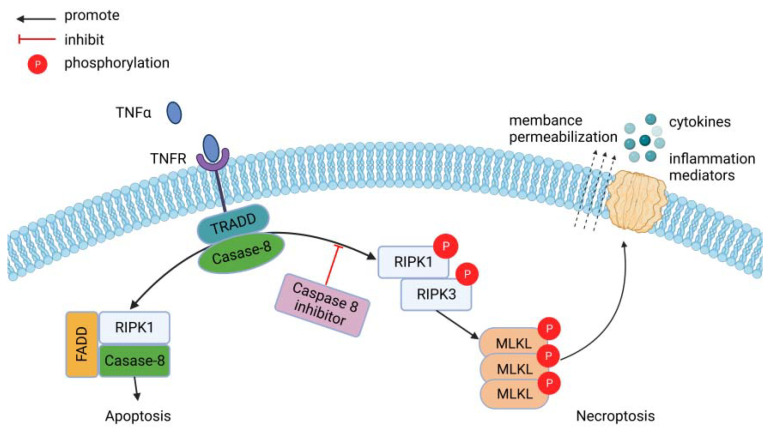
Simplified version of necroptosis signal transduction events downstream of tumor necrosis factor-α/tumor necrosis factor receptor (TNFα/TNFR) interactions. Soluble TNFα binds TNFR and can trigger the formation of a pro-apoptotic (left, complex I) or pro-necroptotic (right, complex IIb) complex. In the absence of caspase-8, the pro-necroptotic complex IIb forms. After phosphorylation of RIPK1 and RIPK3, RIPK3 phosphorylates MLKL, which subsequently oligomerizes and is thought to be inserted into the cell membrane, forming pores. After cell membrane permeabilization, cytokines and intracellular contents are relapsed into the extracellular environment. Figure created through BioRender.com.

RIPK1 and RIPK3 interact through a common RIP homotypic interaction motif (RHIM) to form necrosome vesicles. This leads to phosphorylation and activation of RIPK1 and RIPK3 [26]. MLKL is phosphorylated by activated RIPK3 and oligomerizes as a result, migrating from the cytoplasm to the cell membrane to form open pores that alter ion permeability and lead to necroptosis [23] (Figure 1). RIPK1 is not the only molecule that activates RIPK3. The TIR structural domain junction containing the RHIM structural domain induces interferon-β (TRIF) and DNA-dependent activator of interferon regulatory factor (DAI) can also interact with RIPK3 to form non-classical necroptosis vesicles and participate in the development of necroptosis. The Toll-like receptor ligands mediate the activation of RIPK3 through TRIF, and some viruses not only bind directly to RIPK3 but also promote the binding of DAI to RIPK3, thus participating in necroptosis [27]. Meanwhile, some recent studies have shown that phosphoglycerate mutase 5 (PGAM5) and Ca MKII could also be involved in necroptosis as downstream molecules of RIPK3 [28,29]. In Fritsch’s study, it was shown that caspase-8 induces TNFα-mediated necroptosis, and caspase-8 is the molecular switch for apoptosis, necroptosis, and pyroptosis. Additionally, caspase-8 can cleave RIPK1, which inhibits the development of necroptosis [30]. The RIPK1/RIPK3/MLKL axis is the classical pathway of necroptosis. Activation of RIPK1 and RIPK3 is required during necroptosis, and both CaMKII and MLKL are thought to be executors of programmed cellular necrosis [31].

4-Hydroxy-2-Nonenal (4-HNE), one of the most toxic aldehydes, is a major secondary product of lipid peroxidation [32]. It has been found that both necrotrophic apoprotein and 4-HNE are upregulated in MIRI. Further studies revealed that 4-HNE perfusion enhanced necroptosis in a time- and concentration-dependent manner, and that 4-HNE bound directly to RIPK1, leading to a reduction in RIPK1 degradation mediated by K48 polyubiquitination of RIPK1, activation of the necroptotic pathway, and promotion of myocardial necroptosis [33]. RIPK3 is rapidly increased in hypoxia/reoxygenation (H/R)-treated cardiomyocytes and is associated with myocyte necroptosis [34]. It has been recently shown that, in the context of MI–RI, elevated RIPK3-mediated endoplasmic reticulum stress leads to increased mPTP opening through XO-mediated ROS production [35]. RIPK3 deficiency inhibits mitochondrial permeability transition pore (mPTP) opening, reduces mitochondrial oxidative stress, and attenuates H/R-mediated necroptosis in cardiomyocytes [36]. Similarly, activation of RIPK3 is thought to underlie the structural and histological abnormalities of tissues caused by post-infarct heart failure due to the promotion of inflammation and necroptosis [37]. Chen Li et al. showed that in vivo experiments in mice with Necrostatin-1 treatment (RIPK1 inhibitor), RIPK3 deletion, and cardiac p62 knockdown demonstrated that RIPK1-RIPK3-dependent myocardial necroptosis increased senescence-associated myocardial susceptibility to I/R injury vulnerability [38].

However, necroptosis is unlikely to be the cause of impaired cardiac function during early reperfusion, according to research by Csaba Horvath et al. It is believed that RIPK3 regulates early reperfusion injury through oxidative stress and mitochondrial activity-related effects rather than cell loss due to necroptosis [39].

## 3. Relationship between Necroptosis and Other Associated Mechanisms of MI–RI

In MI–RI, more than one type of regulatory necrosis often plays a role as a result of the superposition of multiple pathways. Although the different types of regulatory necrosis differ in morphological features, the core difference between them lies in the molecular pathways involved. For example, necroptosis is mediated by the RIPK1/RIPK3/MLKL pathway [40], and ferroptosis is closely associated with pathways responsible for iron or cysteine/glutathione homeostasis [2,41]. Meanwhile, CypD-mediated necrosis is dependent on pathways associated with mPTP opening [42]. Parkin can act as a negative regulator of CypD by directly ubiquitinating CypD, which in turn inhibits necrosis, reduces myocardial I/R injury, and improves cardiac function [43]. 

Research by Csaba Horváth et al. has demonstrated for the first time that early reperfusion inhibits the activation of autophagy and suggests that there may be a positive relationship between autophagy and the necroptotic protein RIPK3. Inhibition of RIPK3 during reperfusion significantly attenuated the loss of plasma membrane integrity but did not improve cardiac function [39]. Autophagy is a protective mechanism in chronic myocardial ischemia, and inhibition of autophagy leads to significant exacerbation of cardiac myocytes in ischemia–reperfusion injury. Conversely, enhancing autophagy has a potential protective effect against ischemia/reperfusion injury in cardiac cells [44]. Necroptosis is considered an upstream inhibitor of autophagy [45], while other studies have shown that overstimulated autophagy initiates the necroptotic pathway; therefore, that necroptosis becomes a subsequent procedural event [46]. It has also been suggested that in acute ischemia–reperfusion injury of the myocardium, inhibitors of two pathways, apoptosis and ferroptosis, exert cardioprotective effects on cardiac I/R injury by regulating mitochondrial function. In contrast, necroptosis is not involved in the pathogenesis of this acute cardiac I/R setting [47]. 

ROS is a crucial component of MI–RI [48], and numerous studies have pointed to a connection between ROS and necroptosis. In NIH 3T3 cells, TNF-induced ROS production is RIPK3-dependent [36], and ROS production has been shown to be inhibited in a variety of RIPK3 or MLKL deficient cell lines [49,50]. A study by Zhang et al. showed a direct link between I/R-mediated RIPK3 activation and ROS production in isolated cardiac myocytes, while the potential mechanisms were shown to include CaMKII activation and a subsequent increase in mPTP opening [45]. However, Tait’s experiments showed that mitochondrial ROSs are not necessary for necroptosis and can be bypassed if caspase-8 is inhibited [51]. Non-mitochondrial-derived ROSs are also associated with TNF-induced necroptosis [52]. Therefore, complementary pathways leading to necroptosis may exist. The above evidence provides strong evidence for the involvement of ROS in necrotic signaling; however, the specific mechanisms of ROS involvement in necroptosis still require further exploration.

Some studies have revealed that MAPK and NF-κB are involved in many biological processes [53,54]. p38MAPK-activated protein kinase 2 (MK2), an effector kinase located downstream of MAPK and NF-κB, phosphorylates RIPK1 directly at residue S321 and inhibits RIPK1 integration into the necrosome, thereby suppressing RIPK1 kinase-dependent apoptosis and necroptosis [55]. There is conclusive evidence that RIPK1 phosphorylates signal transducers and activators of transcription 3 (STAT3), contributing to its interaction with the gene associated with retinoid-IFN-induced mortality-19 (GRIM-19) (a subunit of mitochondrial complex I). This causes GRIM-19 translocation to mitochondria, launching the generation of ROS and ultimately bringing on TNF-induced necroptosis [56]. Additionally, Akt (a serine/threonine kinase)/the nuclear factor erythroid-2-related factor 2 (Nrf2) pathway is essential for regulating ROS production [57]. Akt is involved in the positive regulation of Nrf2 antioxidants. Activated Nrf2 further segregates from kelch-like ECH-associated protein 1 (Keap1) and enters the nucleus to form a dimer with Maf, recognizing and inducing transcription of NQO-1 and HO-1 and attenuating oxidative stress injury [58]. Thus, modulation of the Akt/Nrf2 signaling pathway can reduce ROS production-induced necroptosis.

Rho-associated coiled-coil kinase (ROCK), a serine/threonine kinase regulated by the small GTPase RhoA, is involved in regulating cell migration, proliferation, and survival. During MI–RI, RIPK3 mediates the linkage between Rho proteins and substrates and enhances necrotic cell death by activating the RhoA/ROCK pathway [59]. Activation of the RhoA/ROCK pathway can also enhance oxidative stress, inflammation, endothelial dysfunction, fibrosis, and necroptosis in damaged myocardia after MI–RI [60]. CaMKII can act as a RIPK3 substrate, mediating ischemia and oxidative stress-induced myocardial necroptosis [61]. It has also been shown that RIPK3 enhances oxidative stress-induced myocardial necroptosis through the activation of CaMKII [62]. RIPK3 may act as a molecular switch for necroptosis and apoptotic cells involved in myocardial I/R injury during RhoA/ROCK pathway activation [61]. 

Activation of RIPK3 is accompanied by activation of the JNK pathway and upregulation of Bcl-2 19-kDa interacting protein 3 (BNIP3), a downstream regulator of HIF-1alpha. Blocking the JNK pathway eliminates the deleterious effects of H/R injury on mitochondrial function, energy metabolism, and redox homeostasis. Overexpression of Bnip3 eliminates the protective effect of RIPK3 deficiency on cardiomyocyte survival [36]. In addition, RIPK3 overexpression also activates the NF-κB signaling pathway [63], while N″-(carboxymethyl) lysine (CML) is a major member of the advanced glycation end product (RAGE). CML promotes necroptosis by increasing the phosphorylation of RIPK3 and its downstream proteins [64]. Collectively, this evidence revealed that necroptosis is a new type of cell death. Table 1 summarizes the differences between necroptosis and several other types of regulated cell death. At the same time, the occurrence of necroptosis is often accompanied by a rise in markers of myocardial injury, such as cTnI [65,66,67], CK-MB [68,69], and N-terminal pro-brain natriuretic peptide (NT-pro-BNP) [70]. Further support comes from Rosana’s study showing that major adverse cardiac events (MACE) were associated with higher log copies/mL of SARS-CoV-2, cTnI, and pro-BNP in plasma. An active form of MLKL (phosphorylated MLKL, pMLKL) was also raised in the serum of MACE patients. The presence of necroptosis in the heart was revealed by the elevated pMLKL and cTnI levels [71]. There is further work to be done on necroptosis and novel biological molecules/biomarkers (ST2, GDF-15, syndecan-1).

## 4. Potential Interventions of Necroptosis in Myocardial Ischemia/Reperfusion Injury

### 4.1. Necroptosis Inhibitor

Inhibition of necroptosis has recently been shown to attenuate reperfusion injury after AMI in mice, rats, and pigs [24,72,73,74]. Nec-1 is a potent allosteric inhibitor of RIPK1 kinase activity that reduces myocardial cell death and maintains myocardial structural integrity, thereby inhibiting the reactive fibrotic process during late myocardial ischemia/reperfusion. In animal models of degenerative disease, Nec-1 prevented cell death, including necroptosis and apoptosis. In addition, administration of Nec-1 to donors and recipients improved graft function in Lewi rats after lung transplantation by reducing necroptosis [75]. Liang et al. showed that administration of NEC-1 (0.6 mg/kg) at the beginning of reperfusion significantly reduced the release of serum creatine kinase and downregulation of autophagy 24 h after reperfusion, whereas higher concentrations of NEC-1 (1.8 mg/kg) increased mortality in rats with chronic myocardial ischemia [69]. Preclinical data from a model of MI–RI showed that the brain also undergoes dendritic spine loss [76,77], which prevents it from forming synapses to maintain normal cognition. However, a study by Liao et al. showed that Nec-1 did not inhibit p-RIPK1, p-RIPK3, and p-MLKL in the brains of rats with MI–RI. The three proteins were not upregulated in the brain, and Nec-1 did not interfere with the physiological functions of these proteins, probably due to the blood–brain barrier (BBB). In addition, all doses of Nec-1 effectively reduced hippocampal apoptosis after MI–RI [78]. 

In the rat model of persistent coronary blockage, the cardioprotective effects of the necroptosis inhibitor necrostatin-7 were investigated. Necrostatin-7 pretreatment led to a decrease in the plasma level of NT-Pro-BNP, which suggested that the left ventricular function had improved [79]. Compound 547 is a novel RIPK1 inhibitor that promotes cell viability and minimizes mitochondrial damage by decreasing the expression and activation of the necrotic cell group, RIPK1, RIPK3, MLKL, and CaMKII, but not the mRNA level [80]. Advanced glycation end products (AGEs) can be produced more quickly in prolonged hyperglycemic situations as a result of non-enzymatic interactions between glucose and proteins, lipids, or nucleic acids [81]. In a model of AGE-induced cardiomyocyte injury, AGEs raised RIPK3 expression and aggravated the disruption of CaMKIIδ selective splicing. They also accelerated CaMKII activation, increased oxidative stress, triggered necroptosis, and damaged cardiomyocytes. Pretreatment with the RIPK3 inhibitor GSK′872 increased CaMKIIδA and CaMKIIδB expression but significantly decreased AGE-induced ox-CaMKII, p-CaMKII, and CaMKIIδC expression, suggesting that GSK′872 corrected AGE-induced CaMKIIδ selective splicing disorder in cardiomyocytes and attenuated AGE-induced CaMKII activation. Downregulation of RIPK3 or GSK′872 inhibited CaMKII activation, reduced oxidative stress, attenuated necrosis, and ameliorated cellular injury in cardiomyocytes [82]. 

Nesfatin-1 is a hypothalamic polypeptide produced by the translation of its precursor nuclear histone 2. It plays a key role in the control of water uptake, blood pressure, and glucose homeostasis [83]. Nesfatin-1 is also a novel cardiac peptide, mainly through the RIPK1/RIPK3/MLKL axis and the RhoA/ROCK/RIPK3 signaling pathway, protecting against I/R injury. Nesfatin-1 is able to exert cardioprotective effects against MI/R in a dose-dependent manner. Montesanti G, et al. demonstrated in the same rat I/R model that only high doses of Nesfatin-1 (20 μg/kg) were able to inhibit the expression of RIPK1, RIPK3, MLKL, ROCK1, and ROCK2 proteins, and nesfatin-1 intraperitoneal injection reduced the infarct size by 50% [84]. Later, Sharifi et al. also confirmed that high doses of nesfatin-1 (20 μg/kg) significantly reduced the expression levels of these proteins (RIPK3, MLKL, ROCK1, and ROCK2 proteins, ROS, antioxidant peptide (GSH), and MDA levels), restored superoxide dismutase (SOD) activity, inhibited the elevation of cTnI, creatine kinase myocardial band (CK-MB), and lactate dehydrogenase (LDH) activity, restored ejection fraction (EF) and fractional shortening (FS), reduced myocardial interstitial collagen deposition and scar formation, and inhibited cardiac histopathological damage [25]. However, CaMKII was not measured to determine whether nesfatin-1 could simultaneously affect this pathway. A novel Nec-1 analog, (Z)-5-(3,5-dimethoxybenzyl)2-imine-1-methylimidazolin-4-1 (DIMO), could exert myocardial protective effects by reducing RIPK1 activation, inhibiting the interaction of RIPK1 with RIPK3, and restoring impaired autophagic flow. In the oxygen–glucose deprivation/reoxygenation injury model (OGD/R), a 0.1 μm dose of DIMO reduced LDH leakage and the proportion of PI-positive cells, and a dose of 1 or 2 mg/kg of DIMO reduced cardiomyocyte necroptosis and improved myocardial infarct size, whereas DIMO at a dose of 4 mg/kg was ineffective. In addition, DIMO attenuated myocardial I/R-induced lysosomal injury [85]. 

Resveratrol (RES) is an edible compound found in grape skins and red wine. RES exerts biological and pharmacological functions such as anti-aging, anti-inflammatory, anti-cancer, and cardioprotective effects [86,87]. A study by Hu et al. showed that resveratrol (RES) treated with different concentrations of RES-treated H/R cardiomyocytes revealed a significant downregulation of TNF-α, RIPK1, RIPK3, and p-MLKL/MLKL expression, a decrease in necroptosis, and an increase in cell viability in a dose-dependent manner. In addition, RES ameliorated the enhanced effect of TNF-α on programmed necrosis in myocardial H/R injured cells and confirmed the effect of RES in MI–RI rats [88]. The natural product oleanolic acid derivative, 2-cyano-3,12-dioxooleana-1,9(11)-dien-28-oic acid (CDDO), is a necroptosis inhibitor that inhibits the phosphorylation of RIPK1 and RIPK3 in necrotic cells by targeting Hsp90, thereby blocking the formation of necrosomes. A more active analogue was later discovered, compound 20, which was effective against necroptosis in human and mouse cells. With activity to attenuate TNF-induced systemic inflammatory response syndrome (SIRS) and brain I/R injury after oral administration, this compound can also synergize with other inhibitors of necroptosis, including TAK632 (a RIPK1/3 inhibitor), SZM630 (a RIPK3 inhibitor), Nec-1 (a RIPK1 inhibitor), and NSA (an MLKL inhibitor). They can be used as lead compounds for necroptosis inhibitors in I/R therapy [89]. In mouse L929 cells, we found no protective effect of CDDO against TNF-α and Z-VAD-fmk-induced necroptosis. Zhu et al. showed that polypeptide globular adiponectin treatment attenuated post-hypoxia/reoxygenation cardiomyocyte injury, as evidenced by increased cell viability and reduced LDH release. Immunofluorescence staining and Western blotting results showed that both necroptosis and apoptosis were triggered by H/R and attenuated by globular adiponectin. Moreover, globular adiponectin also attenuated the formation of reactive oxygen species, oxidative stress, and p38 MAPK and NF-B signaling, which are significant contributors to necroptosis and apoptosis [90].

### 4.2. Chinese Medicine

Salvia miltiorrhiza Bunge, a traditional Chinese medicine, is widely used in the treatment of cardiovascular diseases for its efficacy in activating blood circulation and resolving blood stasis [91]. Tanshinone I (TI) is the main lipid-soluble component of Salvia miltiorrhiza [92]. Zhuo et al. showed that TI pretreatment attenuated tert-butyl hydroperoxide (t-BHP) and MIRI-induced necroptosis by inhibiting the expression of p-RIPK1, p-RIPK3, and p-MLKL. TI activates the Akt/Nrf2 pathway and promotes the expression of antioxidant-related proteins such as Akt phosphorylation, Nrf2, NQO-1, and heme oxygenase-1 (HO-1) in t-BHP-stimulated H9C2 cells. Furthermore, TI reduces ROS production and reverses the loss of mitochondrial membrane potential (MMP) to alleviate oxidative stress; therefore, TI preconditioning plays a role in myocardial injury [93]. 

Arctiin is one of the main active ingredients extracted from the dried mature fruits of Burdock burdock, commonly known as burdock seeds, from the family Asteraceae, as an ingredient in Chinese pharmacopoeia [94,95]. Arctiin reduces cellular necroptosis by scavenging ROS and restoring mitochondrial function or targeting RIPK1 and/or MLKL. Notably, arctiin can interact with amino acid residues in RIPK1 or MLKL but not RIPK3. Arctiin significantly reduced myocardial I/R injury (myocardial infarction and creatine kinase release), while decreasing the levels of necroptosis-related proteins (RIPK1/p-RIPK1, RIPK3/p-RIPK3, and MLKL/p-MLKL) in the hearts of I/R-treated rats. In the presence of Arctiin, necrosis and LDH release were attenuated in H/R-treated cardiomyocytes. Additionally, arctiin impaired H/R-induced ROS generation and mitochondrial dysfunction (increased MMP and decreased ATP production). Likewise, arctiin reduced H/R-treated H9C2 cells in reactive oxygen species production and improved mitochondrial function [96]. 

Bauhinia championii (BC) is a well-known folk herb from Taiwan, China, mainly used for the treatment of acute and chronic pain and rheumatoid arthritis [97]. In previous studies, BC was considered to have antioxidant activity and anti-inflammatory potential [98]. According to Chen et al., BC significantly decreased the size of infarcts caused by I/R, as well as the release of myoglobin and oxidation of CaMK. It could also block Na^+^ channels to lessen action potential depolarization and lessen necroptosis, which prevented I/R-caused ventricular arrhythmias and cardiomyocyte death. BC may be a novel possibility for the treatment of myocardial infarction and ventricular arrhythmias because it also inhibits the oxidation of CaMK, which lowers I/R-induced necroptosis [99].

Baicalin is one of the major flavonoids isolated from the dried root of Scutellaria baicalensis. Due to its various pharmacological effects, such as its antioxidant and antitumor effects, it is widely used in the treatment of various diseases in Chinese medicine. Recently, the protective effect of baicalin on myocardial infarction was reported [100]. Baicalin protects neonatal rat cardiomyocytes from hypoxic injury by significantly increasing nitric oxide (NO) levels [101]. Bai et al. showed that baicalin could promote NO production and inhibit cardiomyocyte apoptosis by activating the PI3K-AKT-eNOS signaling pathway, which in turn reduces the myocardial infarct size and significantly improves cardiac function. Baicalin had a protective effect on myocardial microvasculature and promoted NO production and cGMP levels in rats with MI–RI. The results of in vitro experiments showed that baicalin significantly increased the cellular activity and function of cardiac microvascular endothelial cells (CMEC) in cardiac microvascular endothelial cells exposed to H/R, and RIPK3 and p-MLKL blocked CMEC necroptosis [102]. It was found that TNF-α induced phosphorylation of RIPK1 and RIPK3, and MLKL was negatively correlated with transforming growth factor-activated kinase 1 (TAK1) phosphorylation. Inhibition of TAK1 phosphorylation enhanced necroptosis [103]. 

Ginsenosides are the main components of ginseng. They have various pharmacological effects, such as vasodilator, antitumor, antidiabetic, anti-inflammatory, and antioxidant effects. Ginsenoside Rg2 is one of the compounds in the original ginseng triol group [104]. Li et al. showed that ginsenoside Rg2 effectively inhibited the phosphorylation of RIPK1, RIPK3, and MLKL in H/R cardiomyocytes and inhibited the formation of the RIPK1/RIPK3 complex (necrosome). More importantly, Ginsenoside Rg2 significantly increased TAK1 phosphorylation and enhanced TAK1 binding to RIPK1 while inhibiting the formation of necrosomes and ultimately reducing I/R-induced necroptosis [103].

### 4.3. Clinical Medication

Metformin is a classical oral hypoglycemic agent, and its role in other diseases has been extensively studied. In a study of animal models by Li et al., the aging-associated autophagy defect leads to increased I/R-induced myocardial necroptosis. Aggregated p62 forms a complex with RIPK1 and RIPK3, promoting the binding of RIPK1 and RIPK3 and subsequent MLKL phosphorylation. This leads to the development of aging I/R heart necroptosis. Metformin restores autophagy and disrupts the p62–RIPK1–RIPK3 complex, effectively reducing I/R-induced necroptosis in the senescent heart. Impaired autophagosome clearance is one of the cornerstones of I/R-induced necroptosis in cardiac myocytes [38]. However, Techyran et al. showed that in a porcine model (3~4 months of age), intravascular administration of metformin before reperfusion to achieve higher intracoronary plasma levels failed to reduce the infarct size [105]. Recombinant apyrase (AZD3366) significantly attenuated the increase in RIPK1, RIPK3, and P-MLKL after 1 h of reperfusion and significantly inhibited the increase in IL-6 and GSDMD-N (markers of cellular scorching). The above effects were not observed with Tegretol. At 24 h of reperfusion, both drugs attenuated the elevation of these markers to the same extent, and their effects were additive [106]. 

Sevoflurane is a volatile anesthetic widely used in cardiovascular surgery. Sevoflurane postconditioning (SPC) is protective against MI–RI, and its myocardial protective effects are regulated by multiple signaling pathways [107,108]. Recent studies have shown that O-Glc NAc transferase (OGT) inhibits necroptosis by targeting RIPK3 [109]. In a study by Zhang et al., it was shown that SPC promoted O-GlcNAc transferase OGT-mediated O-GlcNAcylation of RIPK3, reduced RIPK3 expression, and ultimately led to a reduction in RIPK3–MLKL complex formation to inhibit I/R-induced necroptosis. SPC also improved cardiac function and significantly reduced hemodynamic parameters. However, it was not determined whether SPC promotes RIPK3 degradation by directly affecting OGT [110]. 

Remifentanil is an ultra-short-acting opioid that has been shown to promote cardioprotective effects in selected animal models and some clinical settings [111]. Oxidative protein damage, p21 levels, IL-8-based proinflammatory signaling, and MLKL activity decrease after remifentanil preconditioning. Simultaneously, remifentanil preconditioning reduces phosphorylation signaling of RIPK3 and MLKL and attenuates hypoxia-induced necroptosis in cardiomyocytes [112]. 

Dexmedetomidine (Dex) is a highly selective α2-adrenoceptor agonist with superior sedative, anxiolytic, analgesic, and anti-sympathetic activity [113]. H9C2 (embryonic rat heart-derived myoblast) cells treated with H_2_O_2_ showed a significant decrease in cell viability, an increase in LDH release, and a significant increase in necroptosis. Dex pretreatment attenuated these H_2_O_2_-induced injuries, significantly increased the expression of H_2_O_2_-induced protein HO-1, and decreased protein RIPK1 and RIPK3 expression. All these protective effects of Dex were reversed by yohimbine hydrochloride (YOH) [114]. According to Chen et al., H/R significantly increased CK-MB, cTnI, TNF-α, IL-1β, and IL-6 levels and significantly decreased cell viability, while H/R dramatically raised the protein levels of RIPK1, RIPK3, MLKL, and high-mobility group box-1 (HMGB1). DEX pre-treatment significantly improved H/R-induced necroptosis, cell injury, and inflammation. Silencing of HMGB1 enhanced the protective effect of DEX pretreatment on myocardium. However, only the role of Dex preconditioning in ameliorating I/R-induced inflammation was investigated, and the relationship between oxidative stress and DEX pre-adaptation also needs to be explored [66]. Furthermore, in an in vivo study, Dex pretreatment inhibited inflammatory responses and reduced myocardial infarct size by activating α2 adrenergic receptors in rats with MI–RI [115,116]. 

Zhang et al. discovered that a high glucose environment increased hypoxia injury-induced apoptosis, necroptosis, oxidative stress, and endoplasmic reticulum stress. They also found that a high glucose environment combined hypoxic damage suppressed the activation of Janus kinase 2 (JAK2)/signal transducers and activators of the transcription (STAT3) signaling pathway. In contrast, Empagliflozin (EMPA) protected against I/R and H/R-induced cardiomyocyte injury by activating JAK2/STAT3 signaling under high-glucose environmental conditions, while downregulation of STAT3 reversed the protective effect of EMPA, indicating that EMPA had a beneficial effect on cardiomyocyte protection [117]. Long-term administration of EMPA reduces myocardial infarct size by activating STAT3 in microvascular endothelial cells [118]. 

Simvastatin is a common drug used in the cardiovascular system. Jones et al. reported that in a mouse model of MIRI, simvastatin pretreatment reduced infarct size and preserved myocardial function [119]. Naseroleslami et al. showed that simvastatin pretreatment attenuates injury in a rat heart graft I/R model by targeting caspase-9 and RIPK1 protein activity. Furthermore, during MI–RI, excess ROS production activates the Rho/ROCK pathway, and Rho kinase can accelerate cytokine-mediated inflammatory responses, exacerbating left ventricular remodeling after myocardial infarction. This pathway can be reversed by simvastatin and simvastatin-containing nanovesicles [120]. The Moerke study sought a selection of medicinal drugs to assess their capacity to influence RIPK1-mediated cell death. The anti-epileptic medicine Phenhydan^®^ was found to be a powerful inhibitor of death receptor-induced necroptosis and apoptosis in their small-scale screen. In further research, Phenhydan^®^ inhibited RIPK1’s early activation in the TNF receptor signaling complex-I. It is possible that Phenhydan^®^ also inhibits TNFR1 signaling as a whole. Phenhydan^®^ inhibited pathophysiologic cell death processes by altering cell membrane function, such as lipid raft formation, and blocking necrosome formation/activation as well as death receptor-induced NF-B signaling. Phenhydan^®^ extended this observation to several cell types and species by inhibiting RIPK1-mediated cell death not only in L929 cells but also in murine NIH3T3, murine HT-29, human HT-29, and U937 cells. Hence, Phenhydan^®^ can act as a potent inhibitor of death receptor-induced necroptosis and apoptosis [34]. Tu et al. discovered that the combination of ponatinib and desferrioxamine reduced MI–RI by simultaneously inhibiting necroptosis and ferroptosis. The combination significantly reduced myocardial infarct size and creatine kinase release and was more effective than single-drug therapy [68].

### 4.4. Enzymes That Regulate Necroptosis

PGAM5, a serine/threonine protein phosphatase localized to the outer mitochondrial membrane, functions as a novel inducer of necroptosis. Zhu et al. revealed that PGAM5 enhances I/R-mediated necroptosis in cardiomyocytes and that PGAM5 expression impairs cardiac function by decreasing cardiomyocyte systolic/diastolic characteristics. At the subcellular level, PGAM5 deficiency boosted the copy number and transcript levels of mitochondrial DNA, restored mitochondrial respiration, reduced the formation of mitochondrial ROS, and stopped aberrant mPTP opening during I/R. Cardiac-specific PGAM5 deletion suppressed cardiac inflammation and decreased the size of myocardial infarctions [121]. A study by She et al. demonstrated for the first time that inhibition of PGAM5 attenuates I/R-induced necroptosis in rat hearts by inhibiting the mitochondrial dynamin-related protein 1 (Drp1). They also found that there is positive feedback between RIPK1 and PGAM5 [40]. In a study by Park et al., both RIPK1 inhibition and RIPK3 knockout did not provide the same level of protection against I/R injury in knockout (KO) of the mitochondrial Ca^2+^ uniporter (MCU-KO) mice hearts as they did in wild-type (WT) mice hearts. This indicates that the lack of protection cannot be explained by upregulation of necroptosis [122]. 

SB-706375 is a selective receptor antagonist of human urotensin-II (hU-II) that can inhibit hU-II-induced aortic contraction in rats. SB-706375 significantly reduced necroptosis in cardiac myocytes by decreasing LDH and CK-MB activity, increasing RhoA activity and U-II receptor (UTR), RIPK3, ROCK1, and ROCK2 protein expression, and decreasing cTnI levels in coronary effluent, according to research by Duan et al. [59]. Aldehyde dehydrogenases 2 (ALDH2) may be a key regulator of high-glucose-induced cardiomyocyte injury, and activation of ALDH2 prevented the onset of fibrosis, apoptosis, and necroptosis in a model of high-glucose-induced primary cardiomyocyte injury [123]. Sarco/endoplasmic reticulum Ca^2+^-ATPase (SERCA) can recirculate calcium from the cytoplasm back to the endoplasmic reticulum. In CMEC that had undergone I/R treatment, SERCA overexpression reduced intracellular calcium excess, inhibited the production of the MCU, and stopped the aberrant opening of the mPTP. At the same time, SERCA overexpression significantly reduced I/R-induced luminal narrowing and vessel wall edema, protecting the microcirculation against I/R injury in a calcium/MCU/necroptosis pathway-dependent manner [124]. 

In I/R-injured hearts, adenosine kinase inhibitors markedly reduced I/R-induced myonodular formation, mitochondrial injury, infarct size, LVEF, and LVFS. Further studies showed that ADK inhibitors suppressed RIPK3 and RIPK3 phosphorylation and cystatins-9, cystatins-8, and cystatins-3 activation, but not cystatins-12 activation. Additionally, ADK inhibitors prevented mPTP opening and CaMKII activation, which are crucial for the protection of cardiomyocytes during I/R [125]. A study by Xiang et al. discovered that the level of mmu circ 000338, a cardiac-necroptosis-associated circRNA (CNEACR), was decreased in cardiomyocytes exposed to H/R and in the hearts of mice with I/R damage. Histone deacetylase (HDAC7) is directly bound by CNEACR in the cytoplasm, preventing it from entering the nucleus. Forkhead box protein A2 (Foxa2), which can repress the RIPK3 gene by attaching to its promoter region, is suppressed less as a result of HDAC7-dependent inhibition of transcription. In addition, RIPK3-dependent necroptotic mortality of cardiomyocytes was reduced by CNEACR-mediated overexpression of FOXA2. CircRNAs like CNEACR, which increase cell survival and enhance cardiac function in the I/R-damaged heart, might thereby regulate the activity of HDACs that are related to cardiomyocyte necroptosis [126]. Mitsugumin 53 (MG53), an E3 ubiquitin ligase, attaches many ubiquitin chains to the K316, K604, and K627 sites of RIPK1 to facilitate RIPK1’s destruction by the proteasome and prevent necroptosis. After reperfusion injury, the production of ROS in the infarct area promotes the interaction between MG53 and RIPK1. Application of N-acetylcysteine (NAC) disrupts the interaction between MG53 and RIPK1 and abolishes the MG53-mediated cardioprotective effect [127].

### 4.5. Other

In a mouse model, microRNA-325-3p protects the heart from I/R injury after myocardial infarction by inhibiting RIPK3 and necroptosis [128]. Two cannabinoid receptors, CB1R and CB2R, regulate cannabinoids in the myocardium. Previous research has demonstrated that activating the CB1R promotes cell death by inducing the p38 and JNK/MAPK signaling pathways [129]. In a study by Zaafan, a tiny non-coding RNA known as microRNA-103 controls the production of the Fas-associated protein with death domain (FADD) gene, which is a negative regulator of necroptosis during MI progression. CK-MB and troponin-I levels in animals treated with miR-103 power inhibitor greatly improved, and the histology of the cardiac tissue was almost normal. Through targeting the FADD/RIPK pathway, miR-103 inhibitor can be a strong cardioprotective drug and a viable MI treatment [65]. In the ethanol-induced myocardial injury model, CB2R agonist (JWH-133) significantly decreased ethanol-induced production of total phosphorylated protein content, which resulted in elevated expression of RIPK1, RIPK3, and MLKL. Because the JWH-133 significantly decreased the ethanol-induced rise in total phosphorylated protein content expression level and protected against ethanol-induced cardiac injury, it is postulated that CB2R may be an upstream regulator in the RIPK1/RIPK3/MLKL signaling pathway [130]. 

According to research by Qiu et al., shock wave therapy (SWT) reduced the expression of RIPK1 and RIPK3 and increased cell survival and cytotoxicity in the H/R model. Moreover, SWT reduced the blockage of autophagic flow in response to H/R damage in the fluorescent-tagged LC3 (tfLC3) test. SWT prevented H/R-induced necroptosis, and this action was mediated via the restoration of autophagic flow [131]. Maslinic acid (MA) dramatically reduced cardiac tissue damage, decreased CK-MB and LDH levels, and decreased infarct size, according to research by Lin Li et al. MA also promoted autophagic flow and inhibited apoptosis and necroptosis [132]. High-intensity interval training (HIIT) decreased lipid peroxidation and infarct area in the rat model. On the other hand, HIIT dramatically increased SOD activity and decreased the expression of major mediators of MI-induced necroptosis (MLKL, RIPK3, and RIPK1). HIIT after MI protected LV function and prevented remodeling by restoring EF and FS. In addition, long-term HIIT significantly reduced myocardial interstitial collagen deposition and scar formation [133]. Therefore, the prevention of necroptotic cardiomyocyte death is a new direction for preventing MI–RI. Table 2 summarizes the role of necroptosis and its inhibitors in different models.

### 4.6. Summary and Outlook

In the next ten years, cardiovascular diseases, including AMI, are expected to continue to be the leading cause of death worldwide. According to extant research, necroptosis plays a crucial role in MI–RI, including cardiac inflammation, myocardial infarct size enlargement, cardiac dysfunction, and adverse cardiac remodeling. The main focus of this paper was on the different ways to inhibit necroptosis, all of which greatly decreased the size of myocardial infarctions, diminished the release of inflammatory mediators, and enhanced cardiac function. As a result, preventing necroptotic cardiomyocyte death is a new strategy for preventing MI–RI. For patients with acute coronary syndrome, multi-target strategic therapy can significantly improve their prognosis, and the combined prevention of myocardial ischemia–reperfusion injury will become the best myocardial protection therapy [134]. More than one form of programmed necrosis generally plays a role in MI–RI, usually as a result of the overlapping of multiple pathways. For instance, activation of the JNK/Bnip3 route [36], the RhoA/ROCK pathway [59], CaMKII [62], NF-κB signaling pathway [63], PGAM5/CypD/mPTP [135], and other pathways occurs, along with activation of the classical necroptosis pathway. Many necroptosis activators and inhibitors have been created, and targeted inhibition of necroptosis can supplement traditional reperfusion strategies and existing treatments. The majority of these inhibitors of necroptosis only inhibit a single pathway; hence, pharmacological therapies for the treatment of AMI patients in the future should be developed using medication combinations or pleiotropic drugs. In addition, most studies targeting necroptosis therapy are based on in vitro experiments or animal models, so it is still necessary to assess the feasibility of using these medications in clinical trials and in vivo. In the meantime, special consideration should be given to off-target consequences of necroptosis-targeted medicines to enhance selectivity and safety. Finally, the therapeutic time window for the application of drug therapy plays a crucial role in the successful treatment of reperfusion injuries. In reperfusion-induced cellularity following prolonged ischemia, death can only be avoided if cardioprotective agents are administered at the beginning of reperfusion, ideally before the coronary arteries are reopened [136]. However, even if the time window of administration should precede reperfusion, further studies are needed to determine how long the protective therapy must be applied to completely prevent myocardial ischemia–reperfusion injury.

## Figures and Tables

**Table 1:** Comparison of different forms of programmed cell death.

Cell Death	Major Mediator	Morphological Characteristics	Inflammation Reaction
Necroptosis	Caspase-8, RIPKI, RIPK3, MLKL, CaMKII, PGAM5	Cellular swelling, Plasma membrane rupture, Organelle dilation, Translucent cytoplasm, Abundant release of DAMP	Yes
apoptosis	Caspase-9/8/3/6/7	Cellular shrinkage, Membrane blebbing, Nucleus fragmentation, Chromatin condensation, Formation of apoptotic bodies, Low release of DAMP	No
Ferroptosis	Fe, active oxvgen	Mitochondrial shrinkage, mitochondrial cristae reduction, mitochondrial membrane density increases, mitochondrial outer membrane rupture	No
Pyroptosis	Caspase-1/4/5/11	Cell membrane pores, Cellular swelling, pyknosis	No
CypD-mediated necrosis	CypD, p53	Loss of plasma membrane integorganelle swelling, massive intracellular vacuoles, lack of nuclear fragmentation, mitochondrial swelling and rupture of OMM	No

DAMP, damage-associated molecular patterns; OMM, outer mitochondrial membrane.

**Table 2:** Summary of studies investigating necroptosis and its inhibitors in different models.

Agent	Model	Cell or (and) Animals	Main Findings	Ref.
4-HNE	in vitro H/R, in vivo 30 min I/4 or 24 h R	H9C2 cells, C57BL/6 mice, ALDH2-Tg mice	4-HNE increased RIPK1, RIPK3, MLKL, and CaMKII expression and activation.	[33]
Metformin	Ripk3^−/−^ mice	Male C57BL/6 mice	Metformin treatment disrupted p62–RIPK1–RIPK3 complexes and effectively repressed I/R-induced necroptosis in aged hearts.	[38]
The inhibition of RIPK3 (GSK′872 or HS-1371)	in vivo 30 min I/4 or 10 min R	Adult male Wistar rats	RIPK3 regulated early reperfusion injury via oxidative stress and mitochondrial activity-related effects rather than cell loss due to necroptosis. RIPK3 inhibition prevented plasma membrane rupture and delayed mPTP opening.	[39]
PGAM5 inhibitor	in vitro 10 h H/4 h R, in vivo 1 h I/3 h R, Knockdown of PGAM5 in H9C2 Cells	H9C2 cells, SD rat	The I/R-treated rat heart increased infarct size, CK release, upregulation of PGAM5, Drp1, p-Drp1-S616, RIPK1, RIPK3, and MLKL. These phenomena were attenuated by inhibition of PGAM5 or RIPK1. In H9C2 cells, H/R treatment elevated the levels of PGAM5, RIPK1, RIPK3, MLKL, Drp1, and p-Drp1-S616 and induced mitochondrial dysfunction. These effects were blocked by inhibition or knockdown of PGAM5.	[40]
Parkin	in vivo 5 min I/1 h R	SD rats, adult C57BL/6 mice	Parkin mediated mitophagy, inhibited necroptosis under oxidative stress, and suppressed mPTP opening by catalyzing the ubiquitination of CypD in necrotic cascades.	[43]
Z-vad (apoptosis inhibitors), Nec-1 and Fer-1	in vivo 30 min I/2 h R	Male Wistar rats	Apoptosis and ferroptosis inhibitors exerted cardioprotective effects through the modulation of mitochondrial function and the inhibition of apoptosis and ferroptosis pathways, whereas necroptosis did not participate in the pathogenesis in this acute cardiac I/R setting.	[47]
SB-706375 (a selective receptor antagonist of hU-II)	in vivo 30 min I/15, 30, 60 min R	Male and female SD rats	SB-706375 significantly inhibited the changes of haemodynamic parameters, reduced LDH and CK-MB activities, and decreased cTnI levels.	[59]
CML, the major member of advanced glycation end products	in vivo 40 min I/24 h R, in vitro 12 h H/4 h R	Wild-type C57BL/6J male mice aged 7–8 wk, neonatal mice aged 1–3 d, homozygous RAGE knockout mice	CML increased the phosphorylation of RIPK3 and downstream proteins through RAGE. RAGE deficiency effectively blocked these effects.	[64]
Nec-1	in vivo 30 min I/12, 24, 48, 72 h R	Male SD rats	Nec-1 might reduce myocardial cell death, LV remodeling, and maintain myoarchitectonic integrity. The administration of Nec-1 (0.6 mg/kg) at the onset of reperfusion significantly reduced the release of CK and downregulation of autophagy. At higher concentrations (1.8 mg/kg), Nec-1 would increase the mortality rate of rats subjected to chronic myocardial ischemia.	[69]
Nec-1	in vivo 30 min I/2 h R	Male Wistar rats weighing between 400 and 500 g	Bax and Bcl-2 are potentially central regulators of apoptosis, necroptosis, and ferroptosis, as they serve as crosstalk between these cell death pathways.	[78]
compound 547	H_2_O_2_ stimulation for 24 h	hfCPCs	Compound 547 decreased the necrotic cell population, RIPK1, RIPK3, MLKL, and CAMKII, without decreasing mRNA levels. It also increased cell viability and reduced mitochondrial damage.	[80]
GSK′872, a specific RIPK3 inhibitor, RIPK3 siRNA transfection	AGEs stimulation for 24 h	The cardiomyocytes were isolated from 1–3-day-old SD rats	AGEs increased the expression of RIPK3, aggravated the disorder of CaMKIIδ alternative splicing, promoted CaMKII activation, enhanced oxidative stress, induced necroptosis, and damaged cardiomyocytes. RIPK3 downregulation or RIPK3 inhibitor GSK′872 can improve the above phenomenon.	[82]
DIMO	in vivo 30 min I/4 h R	Adult male SD rats	DIMO at doses of 1 or 2 mg/kg improved myocardial infarct size. The DIMO 4 mg/kg dose was ineffective. DIMO at a dose of 0.1 μM decreased LDH leakage and the ratio of PI-positive cells followed by OGD/R treatment. DIMO Inhibits RIP1K’s interaction with RIP3K.DIMO attenuated myocardial I/R- induced lysosome injury.	[85]
RES	in vivo 30 min I/24 h R	Male SD rats (8–10 weeks old)	The expressions of TNF-α, RIP1, RIP3, and p-MLKL/MLKL in H/R myocardial cells treated with different concentrations of RES were significantly downregulated.	[88]
CDDO	in vivo 2 h I/24 h R (left middle cerebral artery)	HT-29 cells, L929 cells, TNF-induced SIRS, female C57BL/6 J mice (6–8 weeks old), male SD rats	CDDO blocked necrosome formation by targeting Hsp90 to inhibit the phosphorylation of RIPK1 and RIPK3 in necroptotic cells.	[89]
GAD	in vivo 45 min I/3 h R, in vitro H/R model	Female pregnant SD rats	GAD attenuates ROS production and oxidative damage, inhibits H/R induced MAPK/NF-κB signaling, and promotes antiapoptotic Bcl-2 expression.	[90]
TI	in vivo 30 min I/2 h R	H9C2 cells, SD rat	TI pretreatment attenuated oxidative stress by mitigating ROS generation, reversing MMP loss, inhibiting the expression of p-RIP1, p-RIP3, and p-MLKL, and promoting the expression of antioxidant-related proteins such as phosphorylation of Akt, Nrf2, NQO-1, and HO-1.	[93]
Arctiin	in vivo 1 h I/3 h R, in vitro H/R model	Male SD rats, H9C2 cells	Arctiin decreased myocardial infarct size, CK release, and the levels of RIPK1/p-RIPK1, RIPK3/p-RIPK3, and MLKL/p-MLKL in I/R-treated rat hearts. Arctiin reduced ROS production and improved mitochondrial function in H/R-treated H9C2 cells. Arctiin can interact with RIPK1 or MLKL but not RIPK3.	[96]
BC	in vivo 30 min I/1 h R	Langendorff-perfused C57BL/6JNarl mice	BC reduced myocardial infarct size, myoglobin release, and oxidation of CaMKII, decreased different types of ventricular arrhythmias and action potential depolarization, and inhibited Na^+^ current density without changing the kinetics.	[99]
Baicalin	in vivo 45 min I/6 h R, in vitro H/R model	Male Wistar rats	Baicalin promoted the production of NO and cGMP, inhibited myocardial cell apoptosis, improved cardiac function, and decreased the myocardial infarction area in the in vivo IR model. Baicalin suppressed the protein expression of RIPK1, RIPK3, and p-MLKL to interrupt CMEC necroptosis and improved cell activity and function in the in vitro H/R model.	[102]
Rg2	in vivo 30 min I/4 h R, in vitro H/R model	H9C2 cells, Male C57/BL6 mice	Rg2 increased TAK1 phosphorylation and enhanced TAK1 binding to RIPK1 while inhibiting the phosphorylation of RIPK1, RIPK3, MLKL, and RIPK1/RIPK3 complex (necrosome) formation, ultimately reducing MIRI-induced necroptosis.	[103]
Recombinant apyrase, (AZD3366), Ticagrelor	in vivo 30 min I/24 h R	Male SD rats	AZD3366 attenuated the phosphorylation of RIPK1, RIPK3, and MLKL, ultimately reducing necroptosis, inflammation, necrosis, and pyroptosis. The effects of AZD3366 and ticagrelor were additive.	[106]
SPC	in vivo 30 min I/2 h R	Male SD rats	SPC reduced the expression of RIPK3, MLKL, and myocardial infarction size, and improved cardiac function, hemodynamic performance. It also attenuated histopathological changes.	[110]
Remifentanil and other opioids	a model of human cardiomyocytes treated with the hypoxia-mimetic agent cobalt chloride	HCM isolated from the ventricles of the adult heart	Remifentanil preconditioning attenuated hypoxia-induced senescence in HCM, decreased the phosphorylation of RIPK3 and MLKL, and diminished HIF-1α signaling upon cobalt chloride treatment.	[112]
Dex, α2-AR, YOH	H9C2 cells were exposed to various concentrations (100 μM, 500 μM, and 1000 μM) of H_2_O_2_ for 12 h	H9C2 (embryonic rat heart-derived myoblast) cells	H_2_O_2_ decreased cell viability and increased LDH release and necroptotic and apoptotic cell death. Dex preconditioning alleviated these injuries induced by H_2_O_2_. Dex preconditioning increased expression of the protein HO-1 and decreased the expressions of the proteins RIPK1 and RIPK3 induced by H_2_O_2_.	[114]
HMGB1 Knockdown by siRNA	in vitro 6 h H/4 h R	H9C2 (embryonic rat heart-derived myoblast) cells	H/R increased the protein levels of RIPK1, RIPK3, MLKL, CK-MB, cTnI, TNF-α, IL-1β, IL-6, and HMGB. The above indicators were ameliorated via dexmedetomidine preconditioning. Silencing expression of HMGB1 reinforced the protective effects of DEX preconditioning against H/R-induced necroptosis.	[66]
EMPA	in vitro H/R	Rat H9C2 cardiomyocytes	H/R injury induced cell apoptosis, necroptosis, oxidative stress, and endoplasmic reticulum stress. EMPA protected against I/R-induced cardiomyocyte injury by activating JAK2/STAT3 signaling.	[117]
Simvastatin (SIM)	in vivo 45 min I/R	Male Wistar rats	MI–RI gives rise to initiation of the Rho/ROCK pathway, which was reversed by SIM and nano-niosomes containing SIM.	[120]
Phenhydan^®^	all cells were cultured in a humidified 5% CO2 atmosphere	L929, NIH3T3, HT-29, U937, and Jurkat cells, C57BL/6	Phenhydan^®^ treatment suppressed phosphorylation and activation of RIPK1 (p-S166), RIPK3 (p-S227 in human and p-T231/S232 in murine cells), and MLKL (p-S358 in human and p-S345 in murine cells) in these cells.	[34]
Combination of ponatinib with deferoxamine	in vivo 1 h I/3 h R, in vitro 10 h H/4 h R	H9C2 cells, Male SD rats.	The combination of ponatinib with deferoxamine reduces myocardial infarct size and CK release, and the combination therapy is more efficient than single medication.	[68]
PGAM5	in vivo 45 min I/0–24 h R	Cardiac-specific PGAM5 knockout mice,	Cardiac-specific PGAM5 deletion reduced myocardial infarction area, improved cardiomyocyte mitochondrial function, and attenuated cardiac inflammation. Genetic ablation of PGAM5 sustained myocardial function upon I/R injury.	[121]
ALDH2	high glucose-induced cardiomyocyte injury	SD rats	Activation of ALDH2 prevented the happening of fibrosis, apoptosis, and necroptosis in the high-glucose-induced primary cardiomyocyte injury model. The protective effects were related to the inhibition of oxidative stress and inflammation and the changing of MMP14 and TIMP4.	[123]
AAV9-mediated SERCA overexpression	in vivo 45 min I/4 h R	Male C57BL/6J mice	Overexpression of SERCA reduced luminal stenosis and vascular wall edema, attenuated intracellular calcium overload, suppressed MCU expression, and prevented the abnormal opening of mPTP.	[124]
the ADK inhibitor ABT-702 intraperitoneally injected AAV9 (adeno-associated virus)-ADK-shRNA	in vivo 30 min I/4 and 24 h R	C57BL/6 mice	ABT-702 reduced the phosphorylation of RIPK3, MLKL, CaMKII, and infarct size and suppressed the activation of caspase-9, caspase-8, and caspase-3 but not caspase-12. It also prevented the opening of the mPTP in I/R-injured hearts.	[125]
mmu_circ_000338, a CNEACR	in vivo I/R, in vitro H/R	Mice	CNEACR can regulate the cardiomyocyte-necroptosis-associated activity of HDACs, promote cell survival, and improve cardiac function in I/R-injured hearts.	[126]
MG53	in vivo 40 min I/R, in vitro H/R	Adult wild-type, mg53^−^/^−^, and tPA-MG53 mice, hiPSCs-derived cardiomyocytes	MG53 suppressed the activation of RIPK1, RIPK3, and MLKL. Upon injury, the generation of ROS in the infarct zone of the hearts promoted interactions between MG53 and RIPK1.The application of NAC disrupted the interaction between MG53 and RIPK1 and abolished MG53-mediated cardioprotective effects.	[127]
ethanol, CB2R agonists	Ethanol induced myocardial injury	Male C57BL/6J mice	Chronic ethanol exposure induced myocardial injury. Nec-1 alleviates necroptosis and ethanol-induced myocardial injury, and CB2R agonists reduced the phosphorylation of RIPK1, RIPK3, and MLKL.	[130]
SWT	in vitro 5 h H/12 h R	HL-1 cells (HL-1 cardiomyocytes, a cardiac cell line derived from the AT-1 mouse atrial myocyte tumor lineage)	SWT increased cell viability and cytotoxicity in the H/R model, decreased RIPK1, RIPK3, and Beclin1 expression, decreased ROS production, and decreased the ratio of LC3-II/LC3-I following H/R. In the tfLC3 assay, the SWT provoked a decrease in the cumulative autophagosome abundance.	[131]
MA	in vivo 30 min I/R, in vitro H/R	Male SD rats, H9C2 cells	MA alleviated myocardial tissue injury, downregulated CK-MB and LDH levels, and reduced infarct size by promoting autophagic flux.	[132]
HIIT	in vivo 30 min I/8 weeks R	Male Wistar rats	HIIT decreased the expression of MLKL, RIPK3, and RIPK1, reduced lipid peroxidation and infarct size, improved endogenous antioxidant system and heart function, and restored SOD activity. Long-term HIIT decreased MDA levels. HIIT preserved left ventricular function and prevented remodeling by restoring EF and FS. Long-term HIIT reduced the interstitial collagen deposition and scar formation in the myocardium.	[133]

Special note: In vivo I/R models without special remarks in the above table are all LAD. H, hypoxia; I, ischemia; R, reperfusion; ALDH2-Tg, aldehyde dehydrogenase 2-transgenic; 4-HNE, 4-hydroxy-2-nonenal; mPTP, mitochondrial permeability transition pore; SD, Sprague-Dawley; PGAM5, phosphoglycerate mutase 5; CK, creatine kinase; Drp1, dynamin-related protein 1; Nec-1, Necrostatin-1; Fer-1, Ferroptosis-1; hU-II, human urotensin-II; LDH, lactate dehydrogenase; CK-MB, creatine kinase-myocardial band; cTnI, cardiac troponin I; RAGE, receptor for advanced glycation end product; CML, Ne-(carboxymethyl) lysine; LV, left ventricle; H_2_O_2_, hydrogen peroxide; hfCPCs, human foetal cardiomyocyte progenitor cells; AGEs, advanced glycation end products; DIMO, novel Nec-1 analog (Z)-5-(3,5-dimethoxybenzyl)2-imine-1-methylimidazolin-4-1; OGD/R, oxygen–glucose deprivation/reoxygenation; RES, resveratrol; CDDO, triterpenoid compound bardoxolone; GAD, globular adiponectin; ROS, reactive oxygen species; TI, Tanshinone I; MMP, mitochondrial membrane potential; Nrf2, nuclear factor erythroid 2-related factor 2; NQO-1, quinone oxidoreductase-1; HO-1, heme oxygenase-1; BC, Bauhinia championii; NO, nitric oxide; CMEC, cardiac microvascular endothelial cell; Rg2, Ginsenoside; SPC, sevoflurane postconditioning; HCM, human cardiac myocytes; Dex, dexmedetomidine; α2-AR, an α2-adrenoceptor; YOH, α2-AR antagonist yohimbine hydrochloride; HMGB1, high-mobility group box-1; EMPA, Empagliflozin; SIM, Simvastatin; ALDH2, aldehyde dehydrogenases 2; MMPs, matrix metalloproteinases; TIMPs, tissue inhibitors of matrix metalloproteinases; SERCA, sarco/endoplasmic reticulum Ca2þ-ATPase; MCU, mitochondrial calcium uniporter; CNEACR, cardiac-necroptosis-associated circRNA; HDAC7, histone deacetylase; hiPSCs, human pluripotent stem cells; MG53, mitsugumin 53; NAC, N-acetyl cysteine; CB2R, cannabinoid receptors 2; SWT, shock wave therapy; tfLC3, fluorescent-tagged LC3; MA, maslinic acid; HIIT, high-intensity interval training; SOD, superoxide dismutase; MDA, malondialdehyde; FS, fractional shortening; EF, ejection fraction.

## Data Availability

Not applicable.
